# Relationship Between Low Skeletal Muscle Mass and Arteriosclerosis in Western China: A Cross-Sectional Study

**DOI:** 10.3389/fcvm.2021.735262

**Published:** 2021-10-20

**Authors:** Zhenzhen Li, Xiang Tong, Yao Ma, Ting Bao, Jirong Yue

**Affiliations:** ^1^Health Management Center, West China Hospital/West China School of Medicine, Sichuan University, Chengdu, China; ^2^Department of Respiratory and Critical Care Medicine, West China Hospital/West China School of Medicine, Sichuan University, Chengdu, China; ^3^Department of Geriatrics and National Clinical Research Center for Geriatrics, West China Hospital/West China School of Medicine, Sichuan University, Chengdu, China

**Keywords:** arteriosclerosis, arterial stiffness, baPWV, low of skeletal muscle mass, sarcopenia

## Abstract

**Objectives:** This study explored the prevalence and the correlation between low muscle mass and arteriosclerosis in different gender and age groups, to increase the attention paid to the risk factors of arteriosclerosis in the young and middle-aged population.

**Methods:** This was an analytical, cross-sectional study. Data were obtained from healthy individuals recruited from the Health Management Center of W Hospital. The brachial-ankle pulse-wave velocity was used as an indicator of arteriosclerosis, and a bioelectrical impedance analysis was used to assess the body composition.

**Results:** A total of 36,374 subjects (men, 58.4%; women, 41.6%; mean age, 43.74 ± 12.34 years [range, 18–80 years]) participated in this study. The prevalence of low skeletal muscle mass and arteriosclerosis was 17.7 and 53.1%, respectively, in all subjects. Low skeletal muscle mass was significantly associated with arteriosclerosis (OR: 1.435, 95% CI: 1.343–1.533, *P* < 0.001) in all subjects, and the association remained significant in young age (OR: 1.506, 95% CI: 1.353–1.678, *P* < 0.001), middle-age (OR: 1.329, 95% CI: 1.195–1.479, *P* < 0.001), and old age (OR: 1.676, 95% CI: 1.191–2.358, *P* = 0.003), and also significant in men (OR: 1.559, 95% CI: 1.396–1.740, *P* < 0.001) and women (OR: 1.266, 95% CI: 1.143–1.401, *P* < 0.001).

**Conclusions and Implications:** Our results show that the prevalence of low muscle mass and arteriosclerosis is high in the general population, even among middle-aged people and young people, and confirmed that there is a significant independent association between low skeletal muscle mass and arteriosclerosis in all subjects and in different age and gender subgroups.

## Introduction

It has long been recognized that aging is associated with gradual changes in body composition and unfavorable metabolic alterations. The accumulation of fat and loss of lean muscle mass are important changes that occur in adults as they age. Generally, muscle mass decreases by 3–8% every 10 years after 30 years of age (1), and muscle strength, the primary component of body function, decreases by 1–2% every year after 50 years of age (2, 3). Sarcopenia is a syndrome characterized by low skeletal muscle mass and strength, which can lead to undesirable health consequences, including physical disability, decreased quality of life, and an increased risk of mortality. A conservative estimate of the prevalence of clinically relevant sarcopenia is that the syndrome currently affects >50 million individuals, and this number is projected to exceed 200 million in the next 40 years (4). The gradual decrease in the skeletal muscle mass is the primary factor that contributes to sarcopenia, and is also the main feature and important pathophysiological change in sarcopenia. A muscle mass loss of >40% is associated with death, and skeletal muscle loss can lead to reduced strength and functional limitations and/or disability (5). However, the pathogenesis of sarcopenia has not yet been fully elucidated. Additionally, sarcopenia may be associated with reduced exercise levels, weakened neuromuscular function, aging-related hormonal changes (including insulin), pro-inflammatory cytokine levels, muscle cell apoptosis, and genetic and nutritional factors (6). These risk factors and pathogenesis are similar to those of other age-related diseases and those involved in atherosclerosis.

Cardiovascular disease (CVD) is the main cause of death worldwide, and is no longer a disease of old age; the incidence rate among young individuals has increased significantly. Arteriosclerosis is the pathological basis of CVD, which is caused by changes in the structure and function of the media, primarily leading to arterial stiffness, especially in the large arteries (7–9). The pathogenesis of arteriosclerosis is complex and may be related to hemodynamic changes, endothelial damage, abnormal lipid metabolism, and chronic inflammation of blood vessel walls caused by physical and chemical damage, eventually leading to the thickening of the arterial intima, vascular stiffness, and luminal stenosis (10). Arteriosclerosis can be considered as the prodromal stage of atherosclerotic disease, or on the contrary, atherosclerosis can be considered as a form of accelerated arteriosclerosis. Nonetheless, arteriosclerosis is an important manifestation of aging-related, subclinical organ damage, and is also a hallmark of cardiovascular disease (11); as such, arteriosclerosis is an excellent predictive tool with added value in the general population (12), which has been established as an independent predictor of cardiovascular events and cardiovascular mortality.

The loss of skeletal muscle mass and arteriosclerosis are two common phenomena associated with aging among middle-aged and older individuals. Some studies have examined the correlation between low skeletal muscle mass and arteriosclerosis (13–16). However, these studies mainly focused on older subjects, and none included populations with a wider range of ages. Although the age of onset of cardiovascular disease and arteriosclerosis has moved forward, young people pay insufficient attention to arteriosclerosis and lack an understanding of the correlation between low muscle mass and arteriosclerosis. This study explored the correlation between low muscle mass and arteriosclerosis in different age groups, to increase the attention paid to the risk factors of arteriosclerosis in the young and middle-aged population, early detection of risk factors, and comprehensive prevention and control measures.

## Materials and Methods

### Participants

All the research subjects in this analytical cross-sectional study were recruited from the Health Management Center of W Hospital. Consecutive participants were recruited between January 1, 2020, and March 31, 2021. The selection criteria were as follows: ability to perform self-care activities of daily living without difficulty or assistance, and willingness to provide informed consent to participate in the research. Individuals with the following comorbidities were excluded: severe malnutrition, history of myocardial infarction, heart failure, stroke, cancer, and severe hepatic or renal dysfunction; the long-term use of corticosteroids and/or diuretics; physical disability (hands, feet, or limbs), diagnosed by the investigators participating in this study, which could affect physical activity or skeletal muscle mass distribution; and weight change >5% in the previous 3 months. All the participants were informed of the purpose and procedures of the study and provided informed written consent. The study protocol was approved by the Biomedical Ethics Committee of the W Hospital (No. 2021-96).

#### Data Collection

A medical history questionnaire was administered to acquire information about the age, sex, hypertension, diabetes, smoking, and alcohol consumption of the participants. The height of the participants was measured without shoes to an accuracy of 0.1 cm, and the weight of the participants wearing light indoor clothes and without shoes was measured to an accuracy of 0.1 kg. The body mass index (BMI) was calculated by dividing the body weight by height squared (kg/m^2^).

The participants assumed a sitting position and after resting for at least 5 min, a mercury sphygmomanometer was used to measure the systolic blood pressure (SBP) and diastolic blood pressure (DBP). The average of two independent blood pressure readings was used, with an interval of 3–5 min between measurements.

After a fast of at least 8 h, a morning blood sample was obtained from the anterior elbow vein and transferred immediately to the central laboratory for analysis. An automated biochemical analyzer was used to measure the fasting blood glucose (FBG) levels. The lipid profiles included total cholesterol, triglycerides, high-density lipoprotein cholesterol, low-density lipoprotein cholesterol (LDL-C), and uric acid.

The skeletal muscle mass was measured by a bio-impedance analysis using an Inbody 570 (BioSpace, Seoul, Korea). The participants stood upright with their arms abducted apart from their trunk and legs spread slightly. Using segmental body composition and skeletal muscle mass, the appendicular skeletal muscle mass index (ASMI) was calculated using the following equation:

ASMI = total limb lean mass/height^2^

Arteriosclerosis was measured by trained personnel using an automated brachial-ankle pulse-wave velocity (baPWV) instrument (Omron Healthcare Co., Ltd., Kyoto, Japan) in accordance with standard procedures in a quiet room with moderate temperature. After the participant rested for 10 min, the measurement was performed with the subject in the supine position. The baPWV, measured through a time-phase analysis, was calculated by estimating the ratio of the distance between the upper chest and the ankle to the time interval between the arm and ankle according to the patient's height (17).

### Definitions

Subjects who exhibited an SBP ≥140 mmHg and/or a DBP ≥90 mmHg during the physical examination (18), or who had been previously diagnosed with hypertension by health care professionals, regardless of whether they were taking antihypertensive drugs at the time of the study, were diagnosed with hypertension.

Subjects with an FBG level ≥7.0 mmol/L during the physical examination or had been previously diagnosed with diabetes by health care professionals, regardless of whether they were using hypoglycemic drugs at the time of the study, were diagnosed with diabetes.

According to the Asia Working Group for Sarcopenia (AWGS) recommendation (19), an ASMI <7.0 kg/m^2^ in men and <5.7 kg/m^2^ in women was classified as “low skeletal muscle mass.” A baPWV <1,400 cm/s was defined as normal peripheral arterial elasticity, and baPWV ≥1,400 cm/s was defined as peripheral arteriosclerosis (20).

### Statistical Analysis

Statistical analyses were performed using SPSS version 21.0 (IBM Corporation, Armonk, NY, USA). The baseline analysis was performed after dividing the subjects into two different subgroups according to the sex and age, according to the levels of SMI, namely “Low muscle mass” and “Normal.” The normality of distributions was tested using the Kolmogorov-Smirnov test. The descriptive data are expressed as numbers and percentages for categorical variables and mean ± standard deviation (SD) for continuous variables. To assess the differences between the groups, *t*-tests were used for ordinal or continuous variables, and the chi-square test for categorical variables.

A multicollinearity diagnostic was conducted to assess the validity of the regression model by calculating the values of tolerance and variance inflation factor (VIF). The values of tolerance >0.1 and VIF <10 were used to indicate the absence of multicollinearity among the dependent variables. The multivariate logistic regression model was performed in all the subjects and different subgroups, such as age groups (age ≤ 40 years, 40 years < age ≤ 65 years, and age >65 years) and gender groups, using three models: (a) crude; (b) adjusted for age and sex; and (c) adjusted for age, sex, BMI, hypertension, diabetes, triglyceride, total cholesterol, HDL cholesterol, uric acid, smoking, and alcohol consumption.

The statistical significance was set at *p* < 0.05.

## Results

### Characteristics of the Study Population

A total of 36,374 subjects (men, 58.4%; women, 41.6%; mean age, 43.74 ± 12.34 years [range, 18–80 years]) participated in this study. The prevalence of low skeletal muscle mass and arteriosclerosis was 17.7 and 53.1% in the overall study population, 18.7 and 30.9% in the youth group (age ≤ 40 years), 15.1 and 67.6% in the middle-aged group (40 years < Age ≤ 65 years), 31.2 and 87.1% in the older group (age >65 years) (*P* < 0.001), 12.1 and 59.2% in the group of men, 25.6 and 44.4% in the group of women (*P* < 0.001). Table 1 summarized the demographic characteristics of all the subjects.

**Table 1:** Demographic characteristics of all subjects.

	**Overall**	**Subgroups for gender**			**Subgroups for age**			
		**Men**	**Women**	***p***	**Young**	**Middle age**	**Old**	***p***
	***n* = 36,374**	***n* = 21,260**	***n* = 15,114**		***n* = 15,772**	***n* = 18,251**	***n* = 2,401**	
Age, years	43.74 ± 12.34	44.27 ± 12.23	43 ± 12.4	<0.001	32.34 ± 4.39	50.1 ± 6.4	70.04 ± 7.3	<0.001
Male, *n* (%)	21,260 (58.4)	/	/	/	8,777 (55.8)	11,000 (60.3)	1,483 (61.8)	<0.001
BMI, kg/m^2^	23.34 ± 3.26	24.54 ± 3.01	21.75 ± 2.88	<0.001	22.85 ± 3.5	23.8 ± 3	23.99 ± 3	<0.001
SMI (kg/m^2^)	/	7.68 ± 0.65	6.03 ± 0.57	<0.001	/	/	/	/
Low skeletal muscle mass, *n* (%)	6,446 (17.7)	2,579 (12.1)	3,867 (25.6)	<0.001	2,944 (18.7)	2,754 (15.1)	748 (31.2)	<0.001
Arteriosclerosis, *n* (%)	19,310 (53.1)	12,595 (59.2)	6,715 (44.4)	<0.001	4,880 (30.9)	12,338 (67.6)	2,092 (87.1)	<0.001
Hypertension, *n* (%)	11,621 (31.9)	8,523 (40.1)	3,098 (20.5)	<0.001	2,793 (17.8)	7,330 (40.2)	1,499 (62.4)	<0.001
Systolic BP, mmHg	120.70 ± 15.0	123.13 ± 14.5	117.28 ± 15	<0.001	116.5 ± 12.3	122.39 ± 15.16	135.3 ± 17.61	<0.001
Diastolic BP, mmHg	73.6 ± 10.7	76.21 ± 10.63	69.94 ± 10	<0.001	70.67 ± 9.6	75.77 ± 11.08	76.45 ± 10.1	<0.001
Diabetes, *n* (%)	2,009 (5.5)	1,597 (7.5)	412 (2.7)	<0.001	150 (1)	1,368 (7.5)	491 (20.4)	<0.001
Fasting glucose, mmol/L	5.16 ± 1.27	5.28 ± 1.43	4.98 ± 0.97	<0.001	4.83 ± 0.83	5.33 ± 1.39	5.99 ± 1.9	<0.001
Triglyceride, mmol/L	1.56 ± 1.39	1.85 ± 1.61	1.16 ± 0.86	<0.001	1.40 ± 1.39	1.7 ± 1.42	1.55 ± 0.99	<0.001
Total cholesterol, mmol/L	4.80 ± 0.93	4.83 ± 0.92	4.75 ± 0.94	<0.001	4.57 ± 0.86	4.97 ± 0.93	4.95 ± 1.02	<0.001
HDL cholesterol, mmol/L	1.34 ± 0.36	1.19 ± 0.29	1.54 ± 0.36	<0.001	1.34 ± 0.36	1.33 ± 0.37	1.39 ± 0.36	<0.001
LDL cholesterol, mmol/L	2.91 ± 0.8	3 ± 0.78	2.79 ± 0.8	<0.001	2.75 ± 0.75	3.04 ± 0.8	3 ± 0.88	<0.001
Uric acid, μmol/L	/	389.5 ± 79	279.62 ± 60.29	<0.001	345 ± 95.5	341.3 ± 86.16	342.6 ± 82.5	<0.001
Smoking, *n* (%)	10,121 (27.8)	9,858 (46.4)	263 (1.7)	<0.001	3,560 (22.6)	5,909 (32.4)	652 (27.2)	0.002
Alcohol consumption, *n* (%)	16,795 (46.2)	15,519 (73)	1,276 (8.4)	<0.001	7,157 (45.4)	8,794 (48.2)	844 (35.2)	0.015

*Values are mean ± SD or valid percentages (n), BMI, body mass index; SMI, skeletal muscle mass index; BP, blood pressure; HDL, high-density lipoprotein; LDL, low-density lipoprotein*.

*Young: Age ≤ 40 years, Middle-age: 40 years < Age ≤ 65 years, Old: Age >65 years*.

Table 2 summarizes the comparisons between the subjects with and without arteriosclerosis. A total of 19,310 (6.8%) subjects were classified as having arteriosclerosis. Comparing to non- arteriosclerosis subjects, arteriosclerosis subjects were significantly older (48.7 ± 12 years vs. 38.1 ± 10 years, *P* < 0.001), male predominant (65.4 vs. 50.6%, *P* < 0.001), higher BMI (23.9 ± 3.2 vs. 22.9 ± 3.2, *P* < 0.001), while lower muscle mass (18.3 vs. 17%, *P* = 0.004), higher values of hypertension (47.5 vs. 14.4%, *P* < 0.001) and diabetes (9.0 vs. 1.6%, *P* < 0.001), and higher levels of fasting glucose (5.4 ± 1.5 vs. 4.9 ± 0.8, *P* < 0.001), systolic blood pressure (126.1 ± 15.8 vs. 114.5 ± 11.2, *P* < 0.001), diastolic blood pressure (77.4 ± 10.8 vs. 69.3 ± 8.8, *P* < 0.001), triglycerides (1.77 ± 1.57 vs. 1.33 ± 1.1, *P* < 0.001), total cholesterol (4.95 ± 0.95 vs. 4.63 ± 0.87, *P* < 0.001), LDL cholesterol (3.02 ± 0.81 vs. 2.79 ± 0.76, *P* < 0.001), uric acid (353 ± 90 vs. 333 ± 90, *P* < 0.001), lower HDL cholesterol (1.31 ± 0.36 vs. 1.37 ± 0.36, *P* < 0.001), and were more likely to smoke (31.7 vs. 23.4%, *P* < 0.001) and consume alcohol (49.6 vs. 42.2%, *P* < 0.001).

**Table 2:** Comparisons between subjects with and without arteriosclerosis.

	**Arteriosclerosis (*n* = 19,310)**	**Normal (*n* = 17,064)**	***P***
Age, years	48.7 ± 12	38.1 ± 10	<0.001
Male, *n* (%)	65.4 (12,631)	50.6(8,629)	<0.001
BMI, kg/m^2^	23.9 ± 3.2	22.9 ± 3.2	<0.001
Low skeletal muscle mass, *n* (%)	3,540 (18.3)	2,906 (17)	0.004
Hypertension, *n* (%)	9,165 (47.5)	2,465 (14.4)	<0.001
Systolic BP, mmHg	126.1 ± 15.8	114.5 ± 11.2	<0.001
Diastolic BP, mmHg	77.4 ± 10.8	69.3 ± 8.8	<0.001
Diabetes, *n* (%)	1,744 (9.0)	265 (1.6)	<0.001
Fasting glucose, mmol/L	5.4 ± 1.5	4.9 ± 0.8	<0.001
Triglyceride, mmol/L	1.77 ± 1.57	1.33 ± 1.1	<0.001
Total cholesterol, mmol/L	4.95 ± 0.95	4.63 ± 0.87	<0.001
HDL cholesterol, mmol/L	1.31 ± 0.36	1.37 ± 0.36	<0.001
LDL cholesterol, mmol/L	3.02 ± 0.81	2.79 ± 0.76	<0.001
Uric acid, μmol/L	353 ± 90	333 ± 90	<0.001
Smoking, *n* (%)	6,121 (31.7)	4,000 (23.4)	<0.001
Alcohol consumption, *n* (%)	9,581 (49.6)	7,214 (42.2)	<0.001

*Values are mean ± SD or valid percentages (n), BMI, body mass index; SMI, skeletal muscle mass index; BP, blood pressure; HDL, high-density lipoprotein; LDL, low-density lipoprotein*.

### Logistic Regression Analysis

A multicollinearity diagnosis revealed a tolerance of 0.034, VIF of 29.382 for cholesterol, and tolerance of 0.043 and VIF of 23.144 for LDL, thus indicating multicollinearity between cholesterol and LDL; therefore, LDL was excluded.

The results of the univariate analysis showed an association between low skeletal muscle mass and arteriosclerosis (OR: 1.075, 95% CI: 1.019–1.135, *P* = 0.008), and the association remained significant after adjustment for age and sex (OR: 1.241, 95% CI: 1.164–1.322, *P* < 0.001). Similarly, the association was significant in the multiple logistic regression model when other potential confounding factors entered the model (OR: 1.435, 95% CI: 1.343–1.533, *P* < 0.001; Table 3).

**Table 3:** Low skeletal muscle mass associated with odds ratio for arteriosclerosis using logistic regression analysis in overall subjects and subgroups.

**Subgroups**	**Model 1**		**Model 2**		**Model 3**	
	**Crude OR [95% CI]**	***P***	**Crude OR [95% CI]**	***P***	**Crude OR [95% CI]**	***P***
Overall	1.075 [1.019–1.135]	0.008	1.241 [1.164–1.322]	<0.001	1.435 [1.343–1.533]	<0.001
Young	0.923 [0.845–1.007]	0.072	1.399 [1.272–1.538]	<0.001	1.506 [1.353–1.678]	<0.001
Middle-age	1.236 [1.130–1.352]	<0.001	1.202 [1.094–1.320]	<0.001	1.329 [1.195–1.479]	<0.001
Old	1.682 [1.265–2.236]	<0.001	1.598 [1.198–2.132]	0.001	1.676 [1.191–2.358]	0.003
Men	1.598 [1.463–1.745]	<0.001	1.362 [1.237–1.500]	<0.001	1.559 [1.396–1.740]	<0.001
Women	1.038 [0.964–1.117]	0.319	1.222 [1.118–1.336]	<0.001	1.266 [1.143–1.401]	<0.001

*Data are presented as odds ratio (95% confidential intervals)*.

*Young: Age ≤ 40 years, Middle-age: 40 years < Age ≤ 65 years, Old: Age > 65 years*.

*Model 1: No adjustment*.

*Model 2: Adjusted by Age and Gender*.

*Model 3: Adjusted by Age, Gender, BMI, Hypertension, Diabetes, Triglyceride, Total cholesterol, HDL cholesterol, Uric acid, Smoking, Alcohol consumption*.

### Subgroup Analysis by Age

For young people (age ≤ 40 years), the results of the univariate analysis showed an insignificant association between low skeletal muscle mass and arteriosclerosis (OR: 0.923, 95% CI: 0.845–1.007, *P* = 0.072), and the association became significant after adjustment for age and sex (OR: 1.399, 95% CI: 1.272–1.538, *P* < 0.001). The association remained significant after adjustment for other potential confounding factors (OR: 1.506, 95% CI: 1.353–1.678, *P* < 0.001; Table 3).

For middle-aged people (40 years < Age ≤ 65 years), the results of the univariate analysis showed a significant association between low skeletal muscle mass and arteriosclerosis (OR: 1.236, 95% CI: 1.130–1.352, *P* < 0.001), and the association was significant after adjustment for age and sex (OR: 1.202, 95% CI: 1.094–1.320, *P* < 0.001). Additionally, the association remained significant after adjustment for other potential confounding factors (OR: 1.329, 95% CI: 1.195–1.479, *P* < 0.001; Table 3).

For older people (age >65 years), the results of the univariate analysis showed a significant association between low skeletal muscle mass and arteriosclerosis (OR: 1.682, 95% CI: 1.265–2.236, *P* < 0.001), and the association was significant after adjustment for age and sex (OR: 1.598, 95% CI: 1.198–2.132, *P* = 0.001). Moreover, the association remained significant after adjustment for other potential confounding factors (OR: 1.676, 95% CI: 1.191–2.358, *P* = 0.003; Table 3).

### Subgroup Analysis by Sex

For men, the results of the univariate analysis showed a significant association between low skeletal muscle mass and arteriosclerosis (OR: 1.598, 95% CI: 1.463–1.745, *P* < 0.001), and the association was significant after adjustment for age (OR: 1.362, 95% CI: 1.237–1.500, *P* < 0.001). The association remained significant after adjustment for other potential confounding factors (OR: 1.559, 95% CI: 1.396–1.740, *P* < 0.001; Table 3).

For women, the results of the univariate analysis showed an insignificant association between low skeletal muscle mass and arteriosclerosis (OR: 1.038, 95% CI: 0.964–1.117, *P* = 0.319). Additionally, the association became significant after adjustment for age (OR: 1.222, 95% CI: 1.118–1.336, *P* < 0.001). Moreover, the association remained significant after adjustment for other potential confounding factors (OR: 1.266, 95% CI: 1.143–1.401, *P* < 0.001; Table 3).

The multiple logistic regression model in the overall subjects showed that, among potential risk factors, age (OR: 1.074, 95% CI: 1.071–1.076, *P* < 0.001), hypertension (OR: 3.166, 95% CI: 2.984–3.359, *P* < 0.001), diabetes (OR: 1.884, 95% CI: 1.630–2.176, *P* < 0.001), triglycerides (OR: 1.104, 95% CI: 1.074–1.135, *P* < 0.001), total cholesterol (OR: 1.163, 95% CI: 1.127–1.200, *P* < 0.001), and uric acid (OR: 1.001, 95% CI: 1.000–1.001, *P* < 0.001) were independently associated with arteriosclerosis, except BMI (OR: 0.978, 95% CI: 0.968–0.988, *P* < 0.001), HDL cholesterol (OR: 0.883, 95% CI: 0.803–0.971, *P* = 0.010), smoking (OR: 0.867, 95% CI: 0.814–0.924, *P* < 0.001), and alcohol consumption (OR: 0.962, 95% CI: 0.901–1.027, *P* = 0.248; Supplementary Table 1).

The factors associated with the odds ratio for arteriosclerosis using logistic regression analysis in the age (Supplementary Table 2) and gender subgroups (Supplementary Table 3) are shown in the Supplementary Table.

## Discussion

This study has a large sample with a large age span (including the entire adult population), and mainly young and middle-aged people. Our results show that the prevalence of low muscle mass and arteriosclerosis is high in the general population, even among middle-aged people and young people, and confirmed that there is a significant independent association between low skeletal muscle mass (assessed according to BIA) and arteriosclerosis (assessed according to baPWV) in all subjects and in different age and gender subgroups.

It is worth noting that in addition to age, several predisposing factors and mechanisms of skeletal muscle mass loss are also believed to be associated with arteriosclerosis, including low levels of physical activity, sedentary lifestyle, chronic inflammatory state, oxidative stress, insulin resistance (IR), and a decline in the testosterone levels (21–27). Age-related chronic low-grade inflammation is an important cause of low muscle mass, which is characterized by elevated levels of tumor necrosis factor-α (TNF-α), C-reactive protein (CRP), and interleukin-6 (IL-6), and increased CRP and IL-6 levels are associated with increased fat levels (28). Inflammatory factors are associated with a decrease in the skeletal muscle mass and strength (29). It is also one of the main signals that induce muscle apoptosis (30). Chronic inflammation leads to programmed cell death (i.e., apoptosis) and hinders muscle protein synthesis, and impaired repair and regeneration are the possible mechanisms of chronic inflammation leading to muscle degradation (31). Chronic inflammation and oxidative stress lead to endothelial dysfunction, collagen and elastin degradation, changes in the composition and hydration state of proteoglycans, and medial calcification, which gradually cause arterial stiffness. Skeletal muscle is not only distributed throughout the exercise system responsible for body functions, but also in various organs, accounting for most of the glucose metabolism in the human body (32). Skeletal muscle is the main organ for glucose homeostasis, and 75% of postprandial glucose uptake is attributed to skeletal muscle. Low skeletal muscle mass may impair glucose homeostasis, reduce insulin sensitivity, and lead to IR, which can increase the blood pressure and blood lipid levels through a series of reactions and contribute to the process of arteriosclerosis. In addition, hyperglycemia and hyperinsulinemia during IR can increase the risk of arteriosclerosis (33).

At present, the evaluation criteria for low skeletal muscle mass and atherosclerosis are not uniform in different studies, and the results are also inconsistent. In a study involving 208 elderly individuals ≥80 years of age, dual-energy X-ray absorptiometry was used to measure the skeletal muscle mass, pace to assess the muscle function, coronary artery calcification score, and endothelial cell function to assess atherosclerosis, and suggested that sarcopenia is associated with atherosclerosis (34). In another study involving a Japanese cohort >55 years of age, the skeletal muscle mass, determined according to BIA and baPWA was used to assess atherosclerosis, and the results revealed that atherosclerosis and skeletal muscle mass have a negative correlation (35). BaPWV is simply measured by wrapping a pressure cuff around the extremities, which is considered to be a relatively brief, non-invasive, and repeatable method for obtaining data on arterial stiffness (36). Pulse-wave velocity (PWV) is regarded as the gold standard measurement for arterial stiffness and an indicator of vascular damage. A previous meta-analysis reported that PWV is an independent predictor of cardiovascular disease, adverse cardiovascular events, and all-cause mortality (37–39). BIA is also a simple, non-invasive, and reproducible method that can be used for large-scale population screening. It can distinguish between fat mass and fat-free mass. It is widely used to measure the skeletal muscle mass and is one of the few diagnostic criteria for muscle diseases.

Some investigators believe that the loss of skeletal muscle mass is related to arteriosclerosis in men, but not in women. In a study involving 496 middle-aged and elderly patients, the cross-sectional area/weight (CSA/BW) of the middle thigh muscle was used to assess sarcopenia, and the carotid artery intima-media thickness (IMT), and baPWV was used to assess atherosclerosis. The results revealed that the thigh muscle CSA/BW was significantly and negatively associated with carotid IMT and baPWV in men, but not in women (13). In another study involving 427 elderly patients, the skeletal muscle mass in the extremities was assessed, and the radial augmentation index (RAI) was used to assess arteriosclerosis. In patients who were men, the skeletal muscle mass was negatively correlated with RAI; however, this association was not obvious among women (40). However, our findings indicate that a low skeletal muscle mass is associated with atherosclerosis in both men and women. The skeletal muscle mass in women is naturally lower than that in men, and the cut-off values for low skeletal muscle mass are different between the sexes. The diagnostic criteria for low skeletal muscle mass in the above study did not distinguish between the sexes; yet, according to the AWGS recommendation (19), an ASMI <7.0 kg/m^2^ in males and <5.7 kg/m^2^ in females was classified as “low skeletal muscle mass” in our study. Using the same low skeletal muscle mass cut-off value in different sexes results in more normal women being defined with low skeletal muscle mass, which may cause no relationship between low skeletal muscle mass and arteriosclerosis in women subjects. Our results are consistent with those in a study reported by Ricardo, which involved 75 subjects and ASMI was dichotomized according to the first quintile for men (8.81 kg/m^2^) and women (7.57 kg/m^2^) (41).

Although there are some studies on the relationship between low skeletal muscle mass and atherosclerosis, most of them only include the older people and lack different age groups, especially young and middle-aged individuals. In modern young people, factors such as high work pressure, fast pace of life, tight schoolwork, staying up late, unscientific diet, and other factors accelerate vascular aging, and CVD is no longer a senile disease. In the recent years, CVD has been trending toward a younger age. The incidence of CVD in people over 25 years of age is gradually increasing, especially among people between 35 and 44 years of age (42). Our research also confirmed that the prevalence of arteriosclerosis in young and middle-aged people is relatively high; nevertheless, this phenomenon has not attracted attention. The increase in sedentary work, changes in lifestyles, and the development of modern transportation have brought convenience to society, greatly reducing the use of labor, and also leading to a decrease in the daily activities, an increasing number of young people have low skeletal muscle, our research found that the prevalence of low skeletal muscle is higher at all ages, unexpectedly, young, and middle-aged people also have higher prevalence; however, people are not aware of this phenomenon. What is more serious is that very little is known about the association between low muscle mass and arteriosclerosis. Therefore, to prevent the occurrence of CVD, it is necessary to pay attention to young and middle-aged individuals, the risk factors need to be detected early, and comprehensive prevention and control is to be exercised.

Among the traditional risk factors for arteriosclerosis, the association between hypertension, diabetes, cholesterol, and arteriosclerosis is significant in all subjects and in different sex or age subgroups, and triglycerides are associated with arteriosclerosis in young and middle-aged people, which is consistent with the hypothesis that increased triglyceride levels favor the development of atherosclerosis (43), however, the association became insignificant in older subjects. The relationship between triglycerides and arteriosclerosis has always been controversial. In the past, atherosclerosis was believed to be a disease characterized by the accumulation of cholesterol instead of triglycerides in the arteries (44). Although most studies point out that triglycerides are directly related to arteriosclerosis, recent studies have suggested that triglycerides are only biomarkers related to arteriosclerosis, and that triglyceride-rich lipoproteins and their residual particles are considered to be one of the main mediators of the link between arteriosclerosis and triglycerides (45). Moreover, the triglyceride levels may fluctuate drastically with diet and exercise; the fasting levels of triglycerides are highly variable, which may depend on the lipid content and the patient's meal time, and population data are biased; therefore, the fasting triglyceride levels are not always positively correlated with atherosclerosis (44, 46). Hypertriglyceridemia is the most difficult lipid disease to evaluate and treat, and is related to several acquired diseases, such as IR. In addition, in a review by Gill et al., the patients with an increased risk of atherosclerotic CVD have a broader spectrum of plasma lipoprotein abnormalities, especially increased triglyceride-rich remnant particles, in which cholesterol (but not triglycerides) content promotes atherosclerosis (43). Therefore, since most of the people included in our study were young and middle-aged people, and this is a retrospective study and no additional data were collected from the older people, such as comorbidities, statins, or other usage, and no more confounding factors can be corrected. We will establish a prospective cohort for the older group to observe the effects of triglycerides on arteriosclerosis in the future.

From the baseline data, the BMI of the low skeletal muscle mass group was lower than that of the normal group. The BMI is currently the most useful obesity measurement index at the population level, and several previous studies have used BMI to define obesity; however, it cannot distinguish between skeletal muscle and fat; as such, it is not a standardized metric to determine overweight. The individuals with a high BMI may not be obese, but have increased skeletal muscle content, while a normal BMI does not indicate the health status, which may be accompanied by a decreased skeletal muscle mass. Several young people blindly pursue weight loss and often aim to lower their BMI, which is also accompanied by low muscle mass, which may increase the risk of arteriosclerosis. As an important part of the human body, the skeletal muscles play an important role in human function and disease occurrence. Therefore, more importance should be attributed to the body composition analysis in the future, and skeletal muscle mass and body fat should be used as the indicators of obesity rather than BMI.

The population in this study was relatively younger and healthier than similar populations reported in previous studies. The reason for this selective bias is that our study site is a physical examination center of the top hospital in China. Most of the health checkups are healthy people, and patients with diseases or serious illnesses are treated in outpatient clinics or local hospitals. However, the association of low skeletal muscle mass and arteriosclerosis remained exit in this sample. Moreover, the demonstration of the association in this lower-risk population offers strong support for the association exit. Greater effects might have been demonstrated in a higher risk population.

The present study had several limitations. The first of which was its cross-sectional design, which cannot be used to determine causality because it is unclear whether skeletal muscle mass loss precedes atherosclerosis or vice versa. Second, we did not consider some specific and potentially relevant factors, such as comorbidities, statin treatment, physical activity, and inability to correct for confounding factors. Third, single-center studies may be inherently biased. As such, further prospective, multicenter study which included more potentially relevant confounder factors is required.

## Conclusions and Implications

The prevalence of low skeletal muscle mass and arteriosclerosis was high, and there was a significant independent association between them in all subjects and different age and gender subgroups. The main clinical advantage of this study is that it improved the awareness of the prevalence and correlation of low muscle mass and arteriosclerosis, confirmed the new risk factors related to CVD, and provided new clinical ideas for the prevention of CVD in young and middle-aged people. This will provide a research foundation for multicenter prospective cohort studies in the future.

## Data Availability Statement

The original contributions presented in the study are included in the article/Supplementary Material, further inquiries can be directed to the corresponding author/s.

## Ethics Statement

The studies involving human participants were reviewed and approved by Biomedical Ethics Committee of the West China Hospital of Sichuan University. The patients/participants provided their written informed consent to participate in this study.

## Author Contributions

JY, ZL, and XT: study concept and design. ZL and YM: acquisition of data. XT and TB: analysis and interpretation of data. ZL and XT: drafting of the manuscript. JY: critical revision of the manuscript for important intellectual content. All authors contributed to the article and approved the submitted version.

## Conflict of Interest

The authors declare that the research was conducted in the absence of any commercial or financial relationships that could be construed as a potential conflict of interest.

## Publisher's Note

All claims expressed in this article are solely those of the authors and do not necessarily represent those of their affiliated organizations, or those of the publisher, the editors and the reviewers. Any product that may be evaluated in this article, or claim that may be made by its manufacturer, is not guaranteed or endorsed by the publisher.
