# Supplementary material for: Relationship Between Low Skeletal Muscle Mass and Arteriosclerosis in Western China: A Cross-Sectional Study
Source: Front Cardiovasc Med. 2021 Oct 20;8:735262. doi: 10.3389/fcvm.2021.735262 (PMC8563701; doi:10.3389/fcvm.2021.735262)
Supplement: Supplementary file 1 [file Data_Sheet_1.docx]

Table S1. Factors associated with odds ratio for arteriosclerosis using logistic regression analysis.

|  | Model 1 | | Model 2 | | Model 3 | |
| --- | --- | --- | --- | --- | --- | --- |
|  | OR [95% CI] | P | OR [95% CI] | P | OR [95% CI] | P |
| Low skeletal muscle mass | 1.075 [1.019-1.135] | 0.008 | 1.241 [1.164-1.322] | <0.001 | 1.355 [1.260-1.457] | <0.001 |
| Age |  |  | 1.089 [1.086-1.091] | <0.001 | 1.074 [1.071-1.076] | <0.001 |
| Gender |  |  | 0.520 [0.496-0.546] | <0.001 | 0.673 [0.621-0.730] | <0.001 |
| BMI |  |  |  |  | 0.978 [0.968 -0.988] | <0.001 |
| Hypertension |  |  |  |  | 3.166 [2.984-3.359] | <0.001 |
| Diabetes |  |  |  |  | 1.884 [1.630-2.176] | <0.001 |
| Triglyceride |  |  |  |  | 1.104 [1.074-1.135] | <0.001 |
| Total cholesterol |  |  |  |  | 1.163 [1.127-1.200] | <0.001 |
| HDL cholesterol |  |  |  |  | 0.883 [0.803-0.971] | 0.010 |
| Uric acid |  |  |  |  | 1.001 [1.000-1.001] | 0.001 |
| Smoking |  |  |  |  | 0.867 [0.814-0.924] | <0.001 |
| Alcohol consumption |  |  |  |  | 0.962 [0.901-1.027] | 0.248 |

Abbreviations: Data are presented as odds ratio (95% confidential intervals).

Model 1: No adjustment;

Model 2: Adjusted by Age and Gender;

Model 3: Adjusted by Age, Gender, BMI, Hypertension, Diabetes, Triglyceride, Total cholesterol, HDL cholesterol, Uric acid, Smoking, Alcohol consumption

Table S2. Factors associated with odds ratio for arteriosclerosis using logistic regression analysis in age groups.

|  | Young | | | | | | | Middle-age | | | | | | | Old | | | | | | |
| --- | --- | --- | --- | --- | --- | --- | --- | --- | --- | --- | --- | --- | --- | --- | --- | --- | --- | --- | --- | --- | --- |
|  | Model 1 | | Model 2 | | Model 3 | | Model 1 | | | Model 2 | | Model 3 | | Model 1 | | | Model 2 | | Model 3 | |  |
|  | OR [95% CI] | P | OR [95% CI] | P | OR (95% CI) | P | OR [95% CI] | | P | OR [95% CI] | P | OR (95% CI) | P | OR [95% CI] | | P | OR [95% CI] | P | OR (95% CI) | P |  |
| Low muscle mass | 0.923 [0.845-1.007] | 0.072 | 1.399 [1.272-1.538] | <0.001 | 1.506 [1.353-1.678] | <0.001 | 1.236 [1.130-1.352] | | <0.001 | 1.202 [1.094-1.320] | <0.001 | 1.329 [1.195-1.479] | <0.001 | 1.682 [1.265-2.236] | | <0.001 | 1.598 [1.198-2.132] | 0.001 | 1.676 [1.191-2.358] | 0.003 |  |
| Age |  |  | 1.086 [1.077-1.095] | <0.001 | 1.076 [1.067-1.086] | <0.001 |  | |  | 1.083 [1.078-1.089] | <0.001 | 1.065 [1.059-1.072] | <0.001 |  | |  | 1.114 [0.867-1.433] | 0.398 | 1.020 [0.988-1.053] | 0.221 |  |
| Sex |  |  | 0.369 [0.342-0.398] | <0.001 | 0.459 [0.407-0.519] | <0.001 |  | |  | 0.639 [0.598-0.682] | <0.001 | 0.831 [0.741-0.932] | 0.002 |  | |  | 1.032 [1.002-1.063] | 0.038 | 1.092 [0.769-1.550] | 0.623 |  |
| BMI |  |  |  |  | 0.985 [0.971 -1.000] | 0.058 |  | |  |  |  | 0.970 [0.955 -0.985] | <0.001 |  | |  |  |  | 0.973 [0.923 -1.025] | 0.303 |  |
| Hypertension |  |  |  |  | 3.085 [2.809-3.388] | <0.001 |  | |  |  |  | 3.274 [3.020-3.549] | <0.001 |  | |  |  |  | 3.575 [2.746-4.654] | <0.001 |  |
| Diabetes |  |  |  |  | 2.166 [1.490-3.148] | <0.001 |  | |  |  |  | 2.121 [1.783-2.522] | <0.001 |  | |  |  |  | 1.482 [1.037-2.118] | 0.031 |  |
| Triglyceride |  |  |  |  | 1.071 [1.032-1.110] | <0.001 |  | |  |  |  | 1.143 [1.096-1.191] | <0.001 |  | |  |  |  | 1.007 [.841-1.206] | 0.937 |  |
| Total cholesterol |  |  |  |  | 1.088 [1.035-1.143] | <0.001 |  | |  |  |  | 1.160 [1.111-1.212] | <0.001 |  | |  |  |  | 1.154 [0.994-1.340] | 0.061 |  |
| HDL cholesterol |  |  |  |  | 0.921 [0.793-1.068] | 0.277 |  | |  |  |  | 0.920 [0.807-1.048] | 0.210 |  | |  |  |  | 0.725 [0.459-1.145] | 0.168 |  |
| Uric acid |  |  |  |  | 1.000 [1.000-1.001] | 0.140 |  | |  |  |  | 1.001 [1.001-1.002] | <0.001 |  | |  |  |  | 0.999 [.997-1.001] | 0.325 |  |
| Smoking |  |  |  |  | 0.958 [0.873-1.052] | 0.370 |  | |  |  |  | 0.830 [0.759-0.907] | <0.001 |  | |  |  |  | 0.760 [0.548-1.053] | 0.099 |  |
| Alcohol consumption |  |  |  |  | 0.927 [0.842-1.019] | 0.117 |  | |  |  |  | 0.931 [0.848-1.022] | 0.131 |  | |  |  |  | 1.390 [1.010-1.913] | 0.043 |  |

Abbreviations: Data are presented as odds ratio (95% confidential intervals).

Young: Age ≤40 years, Middle-age: 40 years < Age ≤ 65 years, Old: Age > 65 years

Model 1: No adjustment;

Model 2: Adjusted by Age and Gender;

Model 3: Adjusted by Age, Gender, BMI, Hypertension, Diabetes, Triglyceride, Total cholesterol, HDL cholesterol, Uric acid, Smoking, Alcohol consumption.

Table S3. Factors associated with odds ratio for arteriosclerosis using logistic regression analysis in gender groups.

|  | Male | | | | | | Female | | | | | |
| --- | --- | --- | --- | --- | --- | --- | --- | --- | --- | --- | --- | --- |
|  | Model 1 | | Model 2 | | Model 3 | | Model 1 | | Model 2 | | Model 3 | |
|  | OR [95% CI] | P | OR [95% CI] | P | OR [95% CI] | P | OR [95% CI] | P | OR (95% CI) | P | OR [95% CI] | P |
| Low muscle mass | 1.598[1.463-1.745] | <0.001 | 1.362[1.237-1.500] | <0.001 | 1.559 [1.396-1.740] | <0.001 | 1.038 [0.964-1.117] | 0.319 | 1.222 [1.118-1.336] | <0.001 | 1.266 [1.143-1.401] | <0.001 |
| Age |  |  | 1.072 [1.069-1.075] | <0.001 | 1.059 [1.056-1.062] | <0.001 |  |  | 1.113 [1.109-1.117] | <0.001 | 1.094 [1.089-1.099] | <0.001 |
| BMI |  |  |  |  | 0.976 [0.964-0.989] | <0.001 |  |  |  |  | 0.977 [0.959-0.995] | 0.011 |
| Hypertension |  |  |  |  | 2.958 [2.759-3.171] | <0.001 |  |  |  |  | 4.003 [3.570-4.488] | <0.001 |
| Diabetes |  |  |  |  | 2.029 [1.731-2.378] | <0.001 |  |  |  |  | 1.782 [1.269 -2.504] | 0.001 |
| Triglyceride |  |  |  |  | 1.095 [1.063-1.128] | <0.001 |  |  |  |  | 1.181 [1.098-1.269] | <0.001 |
| Total cholesterol |  |  |  |  | 1.107 [1.064-1.151] | <0.001 |  |  |  |  | 1.162 [1.101-1.226] | <0.001 |
| HDL cholesterol |  |  |  |  | 0.864 [0.756 -0.988] | 0.033 |  |  |  |  | 0.999 [0.866-1.154] | 0.993 |
| Uric acid |  |  |  |  | 1.000 [1.000-1.001] | 0.298 |  |  |  |  | 1.001[1.000-1.002] | 0.002 |
| Smoking |  |  |  |  | 0.908 [0.852 -0.968] | 0.003 |  |  |  |  | 0.959 [0.706-1.302] | 0.787 |
| Alcohol consumption |  |  |  |  | 0.980 [0.911 -1.054] | 0.583 |  |  |  |  | 0.942 [.0.815-1.088] | 0.418 |

Abbreviations: Data are presented as odds ratio (95% confidential intervals).

Model 1: No adjustment;

Model 2: Adjusted by Age and Gender;

Model 3: Adjusted by Age, Gender, BMI, Hypertension, Diabetes, Triglyceride, Total cholesterol, HDL cholesterol, Uric acid, Smoking, Alcohol consumption.
